# Benchmarking Software for DDA-PASEF Immunopeptidomics

**DOI:** 10.1016/j.mcpro.2025.101492

**Published:** 2025-12-19

**Authors:** Yannic Chen, Annica Preikschat, Annette Arnold, Riccardo Pecori, David Gomez-Zepeda, Stefan Tenzer

**Affiliations:** 1 Helmholtz Institute for Translational Oncology Mainz (HI-TRON Mainz) – A Helmholtz Institute of the DKFZ, Mainz, Germany; 2 Division D191 Immunoproteomics Unit, DKFZ German Cancer Research Center, Heidelberg, Germany; 3 University Medical Center Mainz, Mainz, Germany; 4 Division D150 Immune Diversity, DKFZ German Cancer Research Center, Heidelberg, Germany; 5 Division D190 Immunopeptidomics Platform, DKFZ German Cancer Research Center, Heidelberg, Germany; 6 Research Center for Immune Therapy [Forschungszentrum für Immuntherapie (FZI)], University Medical Center (UMC) of the Johannes Gutenberg University, Mainz, Germany

**Keywords:** immunopeptidomics, DDA-PASEF, software tools benchmark, HLA-bound peptides, database search

## Abstract

Mass spectrometry (MS) is the method of choice for high-throughput identification of immunopeptides, which are generated by intracellular proteases, unlike proteomics peptides that are typically derived from trypsin-digested proteins. Therefore, the searching space for immunopeptides is not limited by proteolytic specificity, requiring more sophisticated software algorithms to handle the increased complexity. Despite the widespread use of MS in immunopeptidomics, there is a lack of systematic evaluation of data processing software, making it challenging to identify the optimal solution. In this study, we provide a comprehensive benchmarking of the most widespread/used data-dependent acquisition-based software platforms for immunopeptidomics: MaxQuant (https://maxquant.org/), FragPipe (https://fragpipe.nesvilab.org/), PEAKS (https://www.bioinfor.com/peaks-software/) and major histocompatibility complexquant. The evaluation was conducted using data obtained from the JY cell line using the Thunder-data-dependent acquisition-parallel accumulation and serial fragmentation method. We assessed each software’s ability to identify immunopeptides and compared their identification confidence. Additionally, we examined potential biases in the results and tested the impact of database size on immunopeptide identification efficiency. Our findings demonstrate that all software platforms successfully identify the most prominent subset of immunopeptides with 1% false discovery rate control, achieving medium to high identification confidence correlations. The largest number of immunopeptides was identified using the commercial PEAKS software, which is closely followed by FragPipe, making it a viable non-commercial alternative. However, we observed that larger database sizes negatively impacted the performance of some software platforms more than others. These results provide valuable insights into the strengths and limitations of current MS data processing tools for immunopeptidomics, supporting the immunopeptidomics/MS community in determining the right choice of software.

## Highlights

• Benchmarking of database search engines for MHC class 1 & 2 peptide DDA-PASEF data.
• Validation of FDR control of search engines through an entrapment-based strategy.
• Large reference database can have a negative impact on numbers of identification.
• PEAKS 11 found most peptides; FragPipe & MHCquant found more non-reference peptides.
• PEAKS, Fragpipe and MHCquant identified similar numbers of non-isobaric SAVs.

## Graphical Abstract

**Figure:**
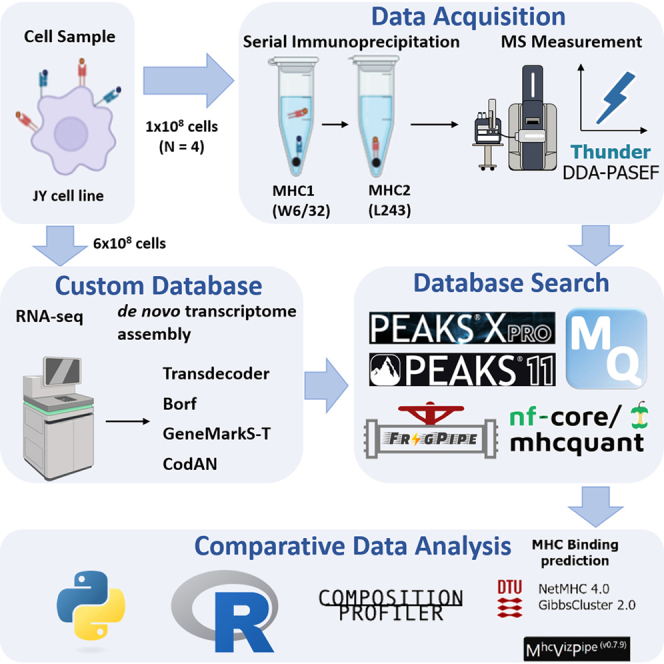

## In Brief

Database searching is a key step in mass spectrometry–based proteomics, identifying peptide sequences from raw spectra. Immunopeptidomics poses unique challenges due to the lack of defined proteolytic specificity. We benchmarked leading database search tools: PEAKS (X Pro and 11), FragPipe, MHCquant, and MaxQuant, on DDA-PASEF immunopeptidomics data. PEAKS 11 achieved the highest total identifications, whereas FragPipe and MHCquant detected the most non-reference peptides, highlighting complementary strengths among search engines.

The major histocompatibility complex (MHC) plays a crucial role in the adaptive immune response by presenting immunopeptides to T-cells, enabling the identification of aberrant cancerous and pathogenic non-self-peptides. MHC are classified into class 1 and class 2, with the former presenting primarily endogenous peptides derived from intracellular proteins while the latter primarily presents exogenous peptides acquired through endocytosis (1, 2). In humans, this protein is termed human leukocyte antigen (HLA) and typically presents peptides of length 8 to 13 amino acids for class 1 and 13 to 25 for class 2 (3, 4). The study of the whole set of immunopeptides, known as the immunopeptidome, is fundamental to many medical research fields, particularly immunotherapy, where disease-specific immunopeptides can be leveraged for targeted treatment (1, 5, 6).

Mass spectrometry (MS) is the method of choice for high-throughput identification of immunopeptides (7). MS techniques infer peptide sequences by comparing observed spectra to hypothetical peptide fragmentation pattern. However, this approach faces challenges due to missing fragmentation from incomplete fragmentations as well as interfering peaks from contaminants/other biological sources in case of multiplexed spectra. To address this, database search algorithms are employed to make informed predictions of peptide identities by limiting the possible identities to known peptide sequences (8). It is important to note that peptides may undergo post-translational modifications (PTMs), which alter the fragment masses and expand the range of potential identities. To account for false positives inherent in this approach, False Discovery Rate (FDR) analysis is used as a quality control measure. FDR employs "decoy" databases containing sequences not present in the sample of interest, to assess the reliability of identifications by quantifying the rate of false "decoy hits" (9).

Immunopeptidomics poses unique challenges for database search engines compared to traditional trypsin-based bottom-up proteomics. In contrast to tryptic digests, immunopeptides are generated by intracellular proteases, necessitating searches without restrictions imposed by proteolytic specificities, dramatically expanding the search space and increasing computational demands (1).

Among various modes of measurement, Data-Dependent acquisition mode (DDA) is the preferred choice for high confidence identifications as it selects the most abundant ions by mass-to-charge ratio (m/z), thereby obtaining high-quality spectra (1, 10).

Recently, tailored acquisition methods on the timsTOF instruments have resulted in high-coverage immunopeptidome profiling of biological samples. These instruments feature a dual trapped ion mobility separation (TIMS) setup, enabling parallel accumulation and serial fragmentation (PASEF) (11). In this approach, peptides are accumulated in packages and then separated based on their ion mobility, significantly enhancing immunopeptide detection (12, 13, 14). However, little attention has been given to evaluating search engines for LC-IMS-MS/MS (Liquid Chromatography Ion Mobility Mass Spectrometer) immunopeptidomics data acquired using DDA-PASEF, a hybrid methodology that integrates DDA with PASEF to achieve high-coverage data acquisition. In addition, many current programs were developed for general proteomics or tested on data from different instruments (*e*.*g*.*,* Orbitrap).

To address this gap, we benchmarked database search algorithms for their performance in identifying immunopeptides from an optimized Thunder-DDA-PASEF acquisition strategy (14). The search engines SEQUEST, MASCOT, and X!Tandem played a foundational role in the development of modern proteomics research. This study, however, focuses on the most current and relevant software available to date. MaxQuant (15), which uses the Andromeda search engine, is one of the most widely used tools for proteomics analysis. MSFragger from FragPipe (16) focuses on speeding up analysis by utilizing fragment ion indexing, making it ideal for searching large search spaces, such as those found in immunopeptidomics. Likewise, PEAKS (17) focuses on *de novo* assisted search, by generating tags for reducing the search space and yielding more robust results. The newly released MHCquant (18), built around the Comet search engine, optimizes workflows for immunopeptidomics by refining result via downstream processes such as rescoring. Analysis of the degree of consensus among the different tools serves as an indicator of the individual result's reliability and quality. Finally, these insights will guide the selection of optimal software tools for specific research contexts.

## Experimental Procedures

### Experimental Design and Statistical Rationale

To generate the benchmarking dataset, MHC class 1 (MHC1p) and MHC class 2 peptides (MHC2p) were enriched from the JY lymphoblastoid cell line using a sequential immunoprecipitation method (See below). Four technical replicates were analyzed via three injections each (n = 12) by LC-IMS-MS employing the Thunder-DDA-PASEF method. For MHC2p analysis, the isolation polygon was modified to accommodate multiply charged ions resulting from longer peptides in the multiply charged ion cloud.

The JY MHC1p and MHC2p datasets were processed using PEAKS X Pro, PEAKS 11, FragPipe (v.18.0 unless otherwise stated), MaxQuant (2.4.4.0), and MHCquant (2.6.0dev). Machine learning-based rescoring methods have been shown to enhance immunopeptide identification from DDA-PASEF data (14, 19). Since PEAKS 11 Studio and Online, and FragPipe include built-in rescoring, these options were activated for this analysis (deep-learning boost and MSBooster (20), respectively). Additional downstream rescoring third party tools for PEAKS X Pro and MaxQuant were not employed in order to compare software performance in their native state.

The heterogeneity in search engines, setting options, and rescoring algorithms across software packages complicates uniform comparison. To mitigate this, variables were standardized where possible (see below), with remaining parameters left at default. To address the quality of matching ambiguous spectra to peptide sequences, 1% peptide-spectrum match (PSM) FDR filtering is employed, unless otherwise stated.

### JY Cell Culture

The EBV (Epstein-Barr virus) transformed human B lymphoblastoid cell line JY (RRID:CVCL_0108) (21) was cultured in RPMI-1640 medium (Sigma-Aldrich, Cat# R8758) supplemented with 10% fetal bovine serum (PAN Biotech, Cat# P40–37100, v/v), 1 mM sodium pyruvate, 2 mM glutamine and 1% of Penicillin/Streptomycin (Sigma-Aldrich, Cat# P4333, v/v). Suspension cell cultures were harvested at 220 x g for 10 min and washed three times with 1 x PBS prior counting and freezing at −80 °C.

### Immunopeptide Enrichment by Immunoprecipitation (IP)

In brief, cell pellets (4 x 10^8^ cells) were defrosted and lysed by adding cold 1% CHAPS (m/v) lysis buffer in PBS, shaking on ice, and sonicating the samples with a Sonicator (Bandelin Sonopuls). Pooled samples were re-distributed into four replicates (1 x 10^8^ cells each) and immunoprecipitated overnight at 4 °C rotating with anti-pan-HLA1 antibody (W6/32, Leinco H263) coupled to cyanogen bromide-activated beads (Cytivia). A second immunoprecipitation of the unbound fraction was performed with anti-HLA-DR antibody (L243, Leinco H261) coupled beads instead. Beads were washed 2 times with 1xPBS and once with H_2_O, followed by seven iterations of elution from beads with 200 μl 0.2% trifluoroacetic acid shaking on ice. Subsequently samples were ultrafiltrated in tubes with 10 kDa molecular weight cut off (Vivacon 500, Hydrosart) and desalted on Oasis HLB plate (Waters) using an elution buffer of 35% acetonitrile, 0.1% trifluoroacetic acid. Finally, samples were lyophilized and dissolved in 15 μl 0.1% formic acid (FA) shortly before LC-MS/MS analysis.

### LC-MS/MS Analysis

Desalted peptides were analyzed by nanoLC-MS using a nanoElute coupled to a timsTOF-Pro-2 mass spectrometer using Thunder-DDA-PASEF for MHC1p (14), with adaptations for MHC2p. Chromatographic separation was performed in a C18 reversed-phase column (Aurora 25 cm × 75 μm ID, 120 Å pore size, 1.7 μm particle size, IonOpticks, Australia). The samples were injected directly into the analytical column and separated using a 47-min gradient, increasing the proportion of phase B (acetonitrile with 0.1% FA (v/v)) to phase A (water with 0.1% FA (v/v)). The gradient started at 2% B, increased to 17% within 23 min, then to 25% in 11.5 min, to 37% in 3.8 min, and to 95% in 3.8 min, and finished with a wash step of 4.9 min at 95% B. Ionization took place using a Captive Spray source, with a capillary voltage of 1600 V, dry gas at 3.0 L/min, dry temperature at 180 °C, and TIMS-in pressure of 2.7 mBar. The MS was piloted using Compass Hystar(v5.1) and timsControl (v.4.0.5) (Bruker). The m/z acquisition range was set at 100 to 2000 Th, and the high-sensitivity detection mode was activated. TIMS was set to a range of 0.65 to 1.75 Vs/cm^2^ with accumulation and ramp time of 300 ms. the number of MS2 frames per cycle was set to three with an overlap of one. The fragmentation intensity threshold was set at 1000 and the target intensity at 20,000. For MHC1p, the Thunder isolation polygon was used to select precursors for fragmentation within the 1/K_0_ *versus* m/z range of peptides with 8 to 13 amino acids, including singly and multiply charged ions (14). For MHC2p, the isolation polygon was modified to include all multiply charged ions and singly charged ions above 550 m/z.

### RNA-Sequencing

RNA was extracted from 6 x 10^6^ JY cells using the AllPrep DNA/RNA Mini kit (QIAGEN, Cat# 80204) and treated with DNase (Invitrogen, Cat# AM 1907). RNA-seq libraries were prepared using Illumina's TruSeq Stranded mRNA Library Prep Kit. Libraries were sequenced with the Illumina NovaSeq 6K PE 100 S4 technology.

### Custom Databases

Unless otherwise stated, the standard reference database used is the UniProt human reference proteome database (retrieved from February 2020) extended by concatenating common contaminant sequences (total sequences = 20,535).

For the *Arabidopsis* entrapment strategy, the final search database was generated by concatenating the above standard reference database with the UniProt *Arabidopsis thaliana* (UP000006548) reference database (retrieved in June 2023) (total sequences = 48,033).

For database size comparison, an updated UniProt human reference proteome was used (retrieved in June 2023). Furthermore, additional protein sequences were included based on RNA-seq data. Total RNA sequencing data from JY cells were quality controlled by Adapter trimming using TrimGalore (0.6.10; https://github.com/FelixKrueger/TrimGalore) (22) and duplicate removal using clumpify (BBMap 39.01) (23). The reads are aligned using STAR (2.7.10 b) (24) with default settings on hg38 assembly and then summarized using featureCounts (Subread 2.0.6) (25) based on University of California Santa Cruz annotation file retrieved from June 2023. Transcripts (including isoforms) with any aligned reads are included in the database (total sequences = 74,261).

*De novo* transcriptome assembly (DNTA) was done in Galaxy. Reads were adapter-trimmed using Trimmomatic (Galaxy Version 0.38.1; https://github.com/timflutre/trimmomatic) (26) and duplicates were not removed. rnaSPAdes (Galaxy Version 3.15.4; https://ablab.github.io/spades/rna.html) (27) on paired-end reads with default settings was used for *DNTA*. Coding sequences were predicted using four softwares: transdecoder (Galaxy Version 5.5.0; https://github.com/TransDecoder/TransDecoder) (28), Borf (v1.3.0; https://github.com/signalbash/borf) (29), GeneMarkS-T (https://exon.gatech.edu/) (30) and CodAN (v1.2) (31), with default settings and translated. The same set of contaminants are always included (total sequences = 602,631).

### DDA Database Search

Raw MS data files from timsTOF (Bruker) mass spectrometer were loaded into the software as individual samples if possible (PEAKS X Pro and PEAKS 11 Online). Option to set different Bioreplicates (FragPipe) and Experiments (MaxQuant) were used and Match between runs was disabled. Unless otherwise stated, the same settings were used for both MHC1 and MHC2 samples. The Precursor Mass Tolerance and Fragment Mass Tolerance were set to 15 ppm and 0.03 da respectively. The peptide length was set to 7 to 25 peptides. For PEAKS X Pro this was not possible and results were manually filtered for 7 to 25 length peptides. Peptide mass range and charges were set to 200 to 5000 and 1 to 4 respectively (FragPipe, MaxQuant and MHCquant). No fixed modification was set and variable modifications includes Oxidation (M), Acetylation (Protein N-term) and Cysteinylation, with a maximum of three variable modifications on peptides. Any build-in rescoring algorithm was kept on (MSbooster/percolator for FragPipe, Deep learning boost for PEAKS 11 Online and percolator/deeplc/ms2pip for MHCquant). Only the Top PSM for each spectrum is sent for rescoring. Enzyme was set to none and digestion mode to unspecific/nonspecific. No extra contaminant Fasta file was provided (however, MaxQuant build-in contaminant was kept on as default). Regarding quantification, MHCquant only worked when the –skip-quantification option was enabled. For FragPipe, MS1 quant was enabled when quantification is needed.

Unless otherwise stated, the Protein and Peptide FDR were set to one or 100%, but the PSM FDR was left at 0.01 or 1% for all but PEAKS X Pro (which doesn’t allow this setting), in which case the 1% PSM FDR was calculated manually (see statistics section). When retrieving all unfiltered candidate PSMs, PSM FDR was also set to one or 100%.

For FragPipe, when specifying different Bioreplicates, a file of the combined PSM result is not generated. This is generated by manually concatenating all psm.tsv files in each “exp” folder together. Unique peptide sequences are obtained by grouping peptide sequences together and keeping the entry with the highest “PeptideProphet.Probability” value. In the same way, a file containing all unique peptidoforms can be generated by using the “Modified Peptide” column instead of the “Peptide” column.

The identification scores used for PEAKS X Pro and PEAKS 11 Online is “X.10LgP” (higher is more confident), for FragPipe is “PeptideProphet.Probability” (higher is more confident), for MaxQuant “PEP” is used (lower is more confident) and MHCquant is “score” (lower is more confident).

### Peptide Binding Prediction

Peptide binding prediction was done using netMHCpan version 4.1 (32) for MHC class 1 and netMHCIIpan version 4.1 (33) for MHC class 2 peptides. For JY cells, the MHC class 1 alleles HLA-A:02:01, HLA-B:07:02 and HLA-C:07:02 were used with a length between 8 to 13 amino acids. For MHC class 2 DRB1:04:04, DRB1:13:01, DRB3:01:01 and DRB4:01:03 were selected with a length of 9 to 22 amino acids. The rank threshold for determining strong and weak binders was kept at default.

### Spectrum ID Comparison

Both spectrum ID and source files are used as unique identifier (except for MHCquant). For PEAKS X Pro, PEAKS 11 Online and Fragpipe software, this information is provided in the psm data file. For Fragpipe specifically, the “Spectrum” column contains the source file and spectrum ID concatenated within a single string. For MaxQuant, the “accumulatedMsmsScans.txt” file is used, where the “PASEF precursor ID” is the equivalent to spectrum ID. MHCquant does not output spectrum ID in their results files. For comparison between itself, the RT and m/z values are used as unique identifier.

### Entrapment Strategy

Database search analysis was conducted as described in the DDA database search section, with the exception of employing the entrapment database and omitting FDR filtering. Subsequently, results were manually filtered to achieve a 1% peptidoform FDR. Matches exclusively associated to decoy accessions were removed. Target hits were defined as peptide hits corresponding to any protein accessions from the standard reference database, while Arabidopsis false positives were classified as identified peptides associated exclusively to the Arabidopsis proteome. For comparative analysis, with the exception of MHCquant, target-decoy results were generated using an analogous approach, with decoy hits serving as false positives and excluding any matches exclusively linked to Arabidopsis protein accessions. MHCquant does not return decoy sequences and thus its 1% peptide FDR filtered results are used instead. Due to MHCquant including PTMs in its calculation its results are analogous to our definition of peptidoform.

### Statistics

The upset plot of shared peptides between software was generated using ComplexUpset (version 1.3.3; https://github.com/krassowski/complex-upset). Sankey graphs were generated using networkD3 (version 0.4.1; https://christophergandrud.github.io/networkD3/).

Amino acid bias is analyzed with the online tool Composition Profiler (34). The background Sample is the aggregate of the identified peptides from all software without removing duplicates. The individual results from each software are compared against this background sample. Significance threshold is set at 0.005 with other settings kept at default.

Spearman correlation coefficient was calculated by ranking the peptides based on the value used to determine their identification confidence (or peptide binding rank in case of binding prediction), unless otherwise stated. Rankings are ordered in a confidence descending order. The pairwise plot is generated using GGally (version 2.2.1; https://ggobi.github.io/ggally/).

For software that did not provide built-in PSM FDR filtering capabilities, manual FDR calculation was performed. This process utilized the unfiltered PSM files generated by the software. FDR was calculated as follows:

$$FDR=\frac{{Decoy}_{CST}}{{Total\_ID}_{CST}}$$

Where ${Decoy}_{CST}$ and ${Total\_ID}_{CST}$ is the number of decoy and total identification remaining after filtering at confidence score threshold CST respectively. The 1% PSM FDR is determined by calculating the FDR at successive confidence score thresholds, moving from the most to least confident scores, and identifying the point where the FDR ≤0.01.

The number of unique 9mers is found by splitting all sequences into all possible 9mers and filtering out duplicates.

## Results

### A Note on Peaks Studio 11 and Peaks Online 11

Bioinformatics Solutions Inc. released PEAKS Studio 11 and PEAKS Online 11 in 2023, succeeding PEAKS X (Pro). Both use identical algorithms, with PEAKS Online 11 optimized for high-throughput processing via multiple nodes (17). We confirmed that both produce identical results given the same inputs and settings. Only minor variations were observed when activating the deep learning boost function (Fig. S1), attributable to the inherent randomness of neural network models across different hardware. Due to this redundancy, only PEAKS 11 Online results were included in further analysis.

### The Choice of Search Engine Affects Peptide Identification from timsTOF Pro Immunopeptidomics Experiments

Software performance was primarily evaluated by the number of unique peptides identified at 1% PSM FDR. Here, we refer to peptides as distinct amino acid sequences and peptidoforms as PTM modified peptide variants.

**Fig. 1:**
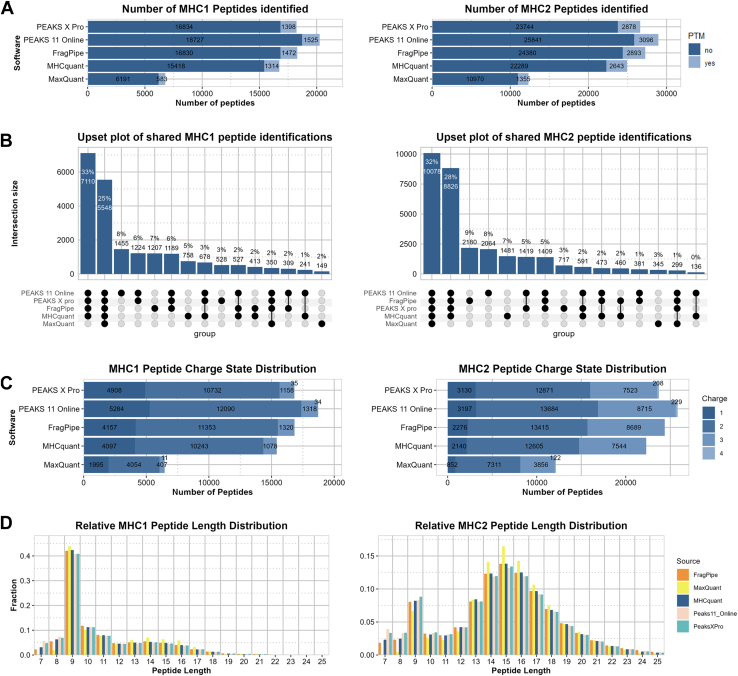
**Comparative analysis of database search engine results**. MHC class 1 results are in the *left* column and MHC class 2 results are in the *right* column. *A*, bar chart showing the number of unique peptides identified by each software. Peptidoforms are included in *lighter blue*. *B*, upset plot showing the intersection size of shared peptide sequences, ordered by intersection size. Only groupings resulting in an intersection size larger than 1% are included. *C*, bar chart summarizing the charge distribution of identified peptides for each software. *D*, column chart showing the relative length distribution of identified peptides for each software. MHC, major histocompatibility complex.

Results (Fig. 1*A*) show that four of the five softwares demonstrate comparable number of identified peptides and peptidoforms. PEAKS 11 Online has the highest number of identifications, with an increase of 6 to 11% for both MHC class 1 and 2 compared to PEAKS X Pro and FragPipe. MHCquant saw a reduction of 6 to 9% compared to PEAKS X Pro and FragPipe. MaxQuant identified the fewest peptides and peptidoforms, finding 60% (MHC class 1) to 50% (MHC class 2) less compared to MHCquant. The PTM distribution (Supplemental Table S1) show overall similar trends across the software except for MHCquant where it has lower identification for cysteinylated MHC1 peptide and Acetylated MHC2 peptides, while also not being able to identify peptides with both acetylation and cysteinylation.

We also assessed the impact of stricter parameters (10 ppm and 0.02 Da and length restriction of HLA1: 8–13mer & max. 1700 Da, HLA2: 15–25mer & max. 2900 Da) on the identification numbers. We observed an overall reduction of identification when using the stricter setting. However, with a fairer comparison of only the number of peptides within the length restriction, we see that for HLA1, the number of 8-13mer identification is increased for PEAKS X Pro and MHCquant, while for HLA2, there is an increased number of identified 15-25mer for Peaks 11 Online, Fragpipe and MHCquant (Supplemental Table S2).

To evaluate identification consistency across software, we compared shared peptide sequences (Fig. 1*B*). 25% of MHC1p and 32% of MHC2p were identified by all software. Excluding MaxQuant, this increases to a total of 58 and 60%, respectively. MaxQuant results show the lowest absolute overlap with other software, primarily due to its lower number of identifications rather than identifying different peptides. Only between 2 to 10% of identified peptides are software specific, indicating general consensus in identifications. To evaluate whether the observed peptide sequence identification consensus extends to the PSM level, we examined whether identical spectrum IDs were assigned to the same peptide sequences. We observed a substantial overlap in spectrum IDs linked to the same sequences between the two PEAKS software versions and MaxQuant. In contrast, most PSMs reported by FragPipe were associated with different spectrum IDs, and more than half of the spectrum IDs it shared with other tools were assigned to different peptide sequences (Supplemental Fig. S2).

### The precursor Charge and Peptide Length Distribution is Largely Conserved Between Search Engines

Beyond sensitivity, assessing peptide identification biases is crucial for validating software performance. To this end, we compared the precursor charge and peptide length distributions across search engines. Charge distributions were largely consistent, with most identification carrying a +2 charge. As expected, MHC class I peptides contained a high fraction of singly charged precursors with relatively few triply charged peptides, whereas MHC class II peptides displayed the opposite trend (Fig. 1*C*). FragPipe and MHCquant did not identify the very rare +4 charged peptides. These results indicate that all tools reproduce the characteristic charge state patterns of MHC-bound peptides, suggesting that no systematic bias in charge assignment is introduced by the search engines. Our results show that singly charged immunopeptides contribute significantly to the total number of identifications (Supplemental Fig. S3). Notably, 76% - 85% of singly charged MHC1 peptides identified in our study were not identified with higher charge states, for MHC2 this proportion is even higher (85%–90%). This indicates that most singly charged peptides would not be identified by acquisition methods targeting only multiply charged ions, consistent with the observations of Gomez-Zepeda *et al*. (2024).

In immunopeptidomics, the length distribution of identified peptides serves as a key indicator of immunopeptide enrichment but also identification performance. Indeed, MHC1p typically have a length of 8 to 13 amino acids with a large predominance of 9-mers (3), while MHC2p extend up to 25 amino acids with a length distribution peaking at 15-mers (in humans) (35). The length distribution is largely consistent across software packages (Fig. 1*D*). The enrichment of 9-mers in the MHC class 1 and length distribution of MHC class 2 samples aligns with expectations and suggests successful immunopeptide enrichment and identification. The presence of 9-mers in the MHC class 2 samples may indicate a co-enrichment of MHC class 1 peptide complexes during immunoprecipitation (36). However, MaxQuant demonstrates reduced efficiency in identifying peptides shorter than nine amino acids. For MHC class 2, this bias is further evidenced by increased identification of longer peptides.

### MHC Binding Prediction indicates a Yield *versus* Specificity Trade-off

Complementing on our analysis of peptide length distributions, we further evaluated software accuracy by leveraging predicted MHC binding affinity. This approach allows for a more comprehensive assessment of software performance in identifying biologically relevant peptides.

**Fig. 2:**
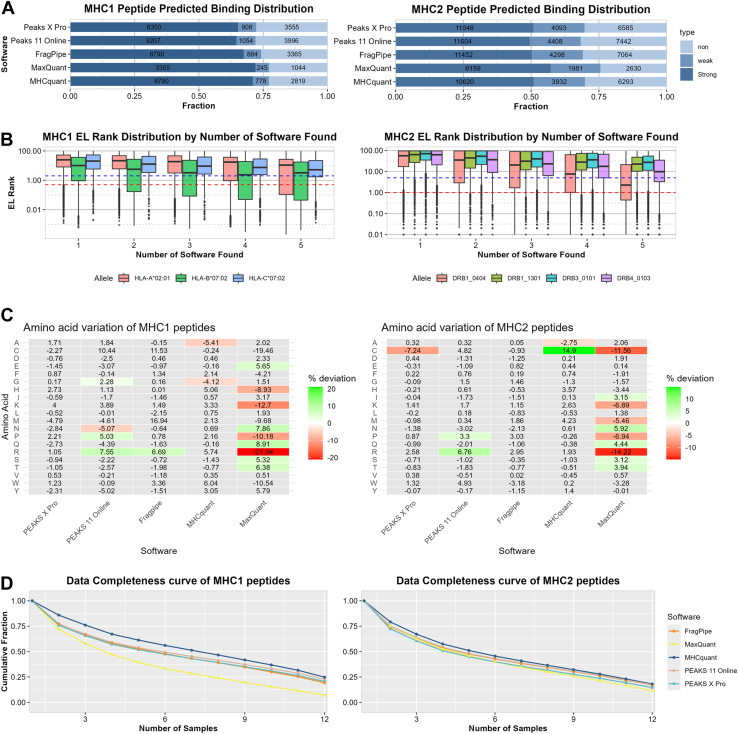
**Analysis on peptide binding strength, amino acid composition and identification consistency**. *A*, bar chart showing the result of netMHCpan (*left*) and netMHCIIpan (*right*) binding prediction results for the result of each software, showing the fraction of strong binders (Rnk_EL ≤ 0.05% MHC class 1 and ≤ 1% class 2), weak binders (Rnk_EL > 0.05% and ≤2% for MHC class 1 and > 1% and ≤5% for class 2) and non-binders (Rnk_EL > 2% for MHC class 1 and > 5% for class 2). *B*, box and whisker plots comparing the rank score distribution of netMHCpan and netMHCIIpan with the number of software the peptide was identified with. *Blue* dashed lines indicate the threshold for weak binders and the *red dashed line* indicates threshold for strong binder. *C*, heatmap of the amino acid composition of the peptides identified by the software. *Gray* indicates not significant based on CompositionProfiler, with a significance threshold of 0.005. Values indicate the percentage difference from the background. *D*, data completeness plot for each software across the 12 samples.

We used netMHCpan (32) and netMHCIIpan (33) to predict binding affinities for identified peptides (Fig. 2*A*). The HLA alleles used are described in the Methods section. For MHC class 1 peptides, PEAKS X Pro and 11 showed similar fractions of predicted binders (0.550 and 0.548 respectively). The other three software packages demonstrated slightly higher fractions, with MHCquant performing best (0.621). For MHC class 2, MaxQuant showed the highest fraction of predicted binder (0.742). The other four software packages performed comparably. PEAKS 11 has slightly lower proportion of predicted binders, but it has the highest total number of predicted binders (10,261 MHC1p and 16,012 MHC2p), followed by FragPipe (9674 MHC1p and 15,750 MHC2p). The increased identification of non-binders by certain software could point to greater sensitivity, since such peptides are expected to be less abundant after purification. However, binding predictions are not fully accurate, and some of these non-binders may in fact represent true ligands that are not recognized by the prediction model or that bind to non-classical HLA molecules (*e*.*g*.*,* HLA-E, HLA-G). Alternatively, they could also arise from contaminants or misassigned spectra.

To investigate whether strong MHC binders are more consistently identified, we examined the relationship between predicted binding affinity and the number of search engines reporting each peptide. As shown in Figure 2*B*, peptides with stronger predicted binding affinities were consistently identified by multiple search engines for both MHC class I and class II across all alleles. Nevertheless, Supplemental Figure S4 shows that non-, weak-, and strong binders are all frequently identified, suggesting that predicted non-binders (perhaps non-MHC peptides) may indeed be present in the measured sample.

### Amino Acid Composition is Largely Conserved Between Search Engines

Parker *et al*. (2021) previously reported that search engines may prioritize different peptides based on their amino acid composition, which would introduce a bias in immunopeptide identification (37). To evaluate if this is the case with the software tested here for DDA-PASEF data, we employed Composition Profiler (34) to quantify significant variations. This software tests whether certain amino acids or properties are enriched or depleted in a dataset compared to a background population. Here, we used the aggregate of all identified peptides as background. This approach provides a robust baseline, capturing the full spectrum of identifications and reflecting software-specific biases in amino acid composition of identified peptides, independently of other factors such as binding-motif specific enrichment. It's important to note that this method introduces another bias, as software identifying more peptides contributes more to the background population.

Analysis of the amino acid distribution of identified peptides revealed largely conserved patterns across search engines. Notably, MaxQuant displayed the greatest number of significant deviations in amino acid frequencies for both MHC class I and II peptides (Figs. 2*C*, Supplemental Fig. S5). Consistently, MaxQuant also showed the most frequent significant differences in broader amino acid properties, such as charge and polarity (Supplemental Table S3). These findings suggest that MaxQuant's peptide identification algorithm introduced the most distinct biases in the amino acid composition of reported peptides compared to other software packages. It must be noted that for MHC class 1, MHCquant has nearly the same number of significantly enriched/depleted properties as MaxQuant. Finally, visualization of motifs using unsupervised Gibbs Clustering by GibbsCluster (https://services.healthtech.dtu.dk/services/GibbsCluster-2.0/) (38) showed no obvious differences. (Supplemental Fig. S6). Altogether, these results indicate that, despite the slight differences, all the programs identified peptides with similar MHC binding motifs.

### Missed Consensus Peptides

To further explore differences between search engines, we defined “consensus peptides” as those identified by at least three database search engines. We then examined the properties of consensus peptides that were not detected by each individual software (Supplemental Fig. S7). Interestingly, a large fraction of the consensus peptides missed by FragPipe for both MHC class I and II are at the shorter end of the length distribution. This appears to be advantageous for MHC class II, as many of these missed consensus peptides are predicted non-binders.

### Reproducibility and Quantification

To compare the reproducibility of the search engines across the measured samples, we generated a data completeness curve (Fig. 2*D*). The result show similar data completeness with MHCquant performing slightly better while MaxQuant slightly worse for MHC1 peptides. We further assessed peptide quantification reproducibility by calculating the coefficient of variation (CV) for each peptide (area for PEAKS X Pro and PEAKS 11 Online and intensity for FragPipe and MaxQuant. MHCquant quantification was skipped due to software issues) across samples and found that MaxQuant exhibited the largest CV distribution, indicating higher variability in peptide quantification (Supplemental Fig. S8*A*).

Finally, we compared peptide quantification between search engines to assess consistency in relative abundance. Supplemental Figure S8*B* shows strong correlations across all software, based on Spearman rank correlation coefficients, demonstrating a high degree of agreement for peptides identified by multiple tools.

### Peptide Identification Scores Correlate Across Software

**Fig. 3:**
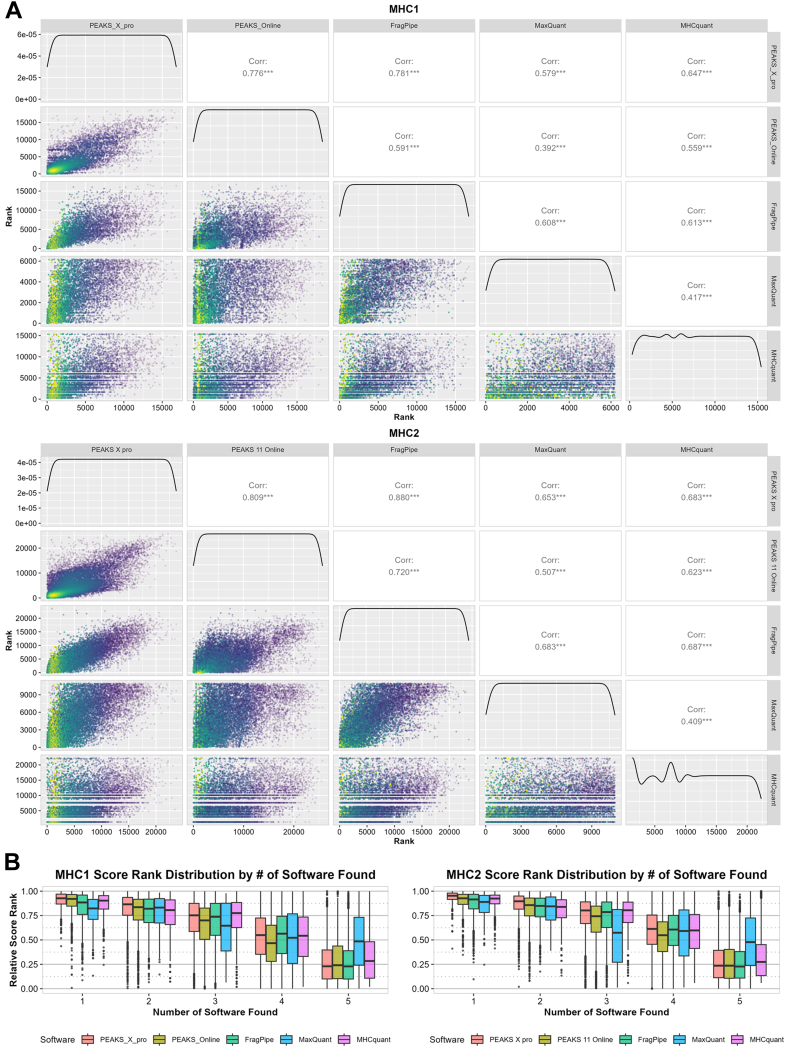
**Analysis of identification confidence score between search engine results**. *A*, pairwise Spearman rank correlation analysis of all software. *Bottom left* section displays scatterplot of ranks of shared peptides between software (x and y axes correspond to the scores of the software indicated in the *top* or *right* header, respectively). *Top right* section returns the respective spearman rank correlation coefficient. Line plots in the center shows the rank distribution of the data. For FragPipe, hyperscore is used instead of the probability due to the lower precision caused by rounding, which leads to many shared ranks at high probabilities. Since hyperscore is used to calculate probability, they are highly correlated, making hyperscore a suitable alternative. The remaining software uses the standard values for rank calculation (*i*.*e*.*,* X.10LgP for PEAKS, PEP for MaxQuant and Score for MHCquant). *B*, Box and whisker plot showing the relative score rank distribution of peptides found by specific number of software. Lower rank indicates a more confident identification.

Comparing the scores of identified peptides between software is crucial for revealing discrepancies in confidence levels and ranking of matches, potentially uncovering algorithmic biases that may be hidden when only considering overlap. However, direct comparison of absolute scores is challenging due to varying scoring methods and the impact of rescoring/boosting, which often results in bimodal score distributions. Therefore, to assess result similarity, we calculated pairwise Spearman correlation coefficients (Fig. 3*A*). Results show moderate to strong correlations between software. PEAKS X pro and FragPipe show the most similar confidence levels with a spearman correlation score of 0.8, while the lowest correlation can be observed between MaxQuant and Peaks 11 Online. Using the relative rankings, we observed that peptide with better ranks are consistently found across multiple software (Fig. 3*B*).

Following the observations in Figure 2*B*, we hypothesized that higher-affinity peptides could more likely persist through the sample preparation and accumulate for better MS identification. To this end, we evaluated the correlation between the Rnk_EL score and each software score rank. This however showed little to no correlation (Supplemental Fig. S9).

These findings underscore the complex interplay between peptide identification algorithms, scoring methods, and biochemical factors in immunopeptidomics, highlighting the need for cautious interpretation of software confidence scores and their relationship to immunopeptide identification.

To ensure clarity, we focus the remaining sections on MHC class 1 peptides. As of writing, MHC one reflects the current interest trend in the immunopeptide research community, allowing for a more manageable scope and a deeper, more consistent evaluation of search engine performance.

### *Arabidopsis* Proteome Entrapment Sequence Indicate No Bias in FDR Control Strategy

FDR filtering using decoys helps mitigate false positives. However, unbiased implementation of this can be difficult. An effective way to assess the robustness of each software’s FDR implementation involves implementing an entrapment strategy with externally supplied entrapment sequences that act as an independent set of false positives to the software generated decoy sequences. This method helps detect potential biases from the software own FDR control implementations and provides a more comprehensive and independent evaluation of software results. Here we used *A*. *thaliana* proteome sequences as entrapment sequence.

In order to compare the performance between the Arabidopsis entrapment strategy and the native target-decoy strategy, a 1% peptidoform FDR cutoff was used for both approaches. We found that a 1% PSM FDR approximates to 1.5 to 3.2% peptidoform FDR (and ∼5% peptide FDR if quantifiable) depending on software (Supplemental Table S4). This shows that peptidoform level FDR acts as a stricter quality control feature compared to PSM FDR.

**Fig. 4:**
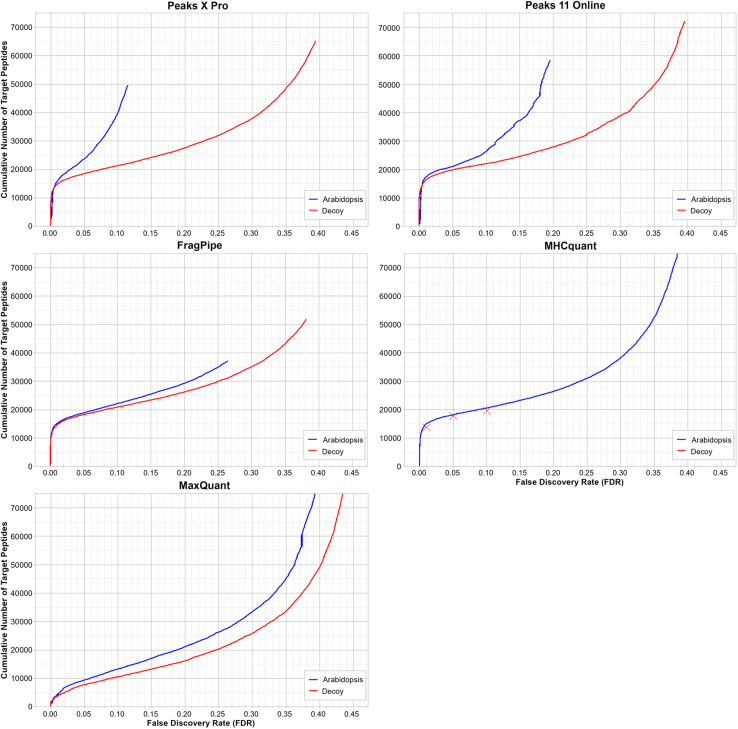
**Identification performance of MHC1 peptides of different software using decoy and entrapment-based FDR control strategy**. Each graph shows the number of target peptide identified at each peptidoform FDR percentage level when using the standard decoy method (*red*) or the entrapment strategy (*blue*). Since MHCquant does not report Decoys, the software was run at 1%, 5% and 10% peptidoform FDR to obtain datapoints for comparison and displayed as “X”. Y-axis limits were set to 75,000. FDR, false discovery rate.

The entrapment strategy yielded higher number of identifications at 1% peptidoform FDR for all software compared to their native target-decoy FDR control. For the software that could be tested, this gap in identification expanded as the FDR increased (Fig. 4). This indicates that decoy hits are not inherently scored worse over the *Arabidopsis* false positive hits, suggesting that the native target-decoy implementations are unbiased and not overestimating FDR filtered identification.

### Database Size Affects Peptide Identification

As we have seen in the previous section, the reference database is a critical parameter in proteomics searches, affecting consistency and accuracy of peptide identifications by influencing FDR filtering and scoring. Here we further investigate this by systematically testing different database size to reveal how software adapts to changing search spaces, exposing strengths and limitations not evident with a single fixed database.

**Fig. 5:**
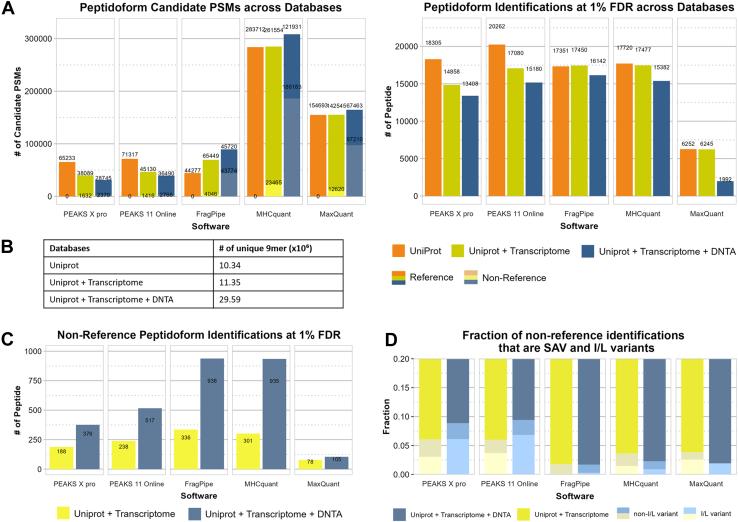
**Comparison of the MHC1 identification results from different software given databases of various sizes originating from UniProt reference proteome, RNA-seq transcriptome data and de-novo transcriptome assembly**. *A*, grouped Stacked bar chart show the total number of identified peptidoform candidates from different software using different databases. Lighter color represents candidates not from UniProt reference database. (For the *right* plot, the non-UniProt reference fraction has been moved to plot C for clarity). *B*, table that shows the complexity of the databases. *C*, bar plot shows the number of non-reference identification at 1% FDR. *D*, Grouped stacked bar plot showing the fraction of non-reference peptide identifications at 1% peptide-spectrum match FDR filtering that are single amino acid variant relative to the UniProt reference database and the fraction of these which are I/L variants (substitution of isoleucine with leucine or *vice versa*.). FDR, false discovery rate.

To simulate real-world research scenarios, we crafted a series of reference databases of varying sizes, based on JY cell line RNA-seq data (see Methods and Fig. 5*B* table). This approach serves a dual purpose: it minimizes the inclusion of irrelevant sequences likely absent in our samples, while simultaneously incorporating non-canonical isoforms and accounting for edits, mutations, and variants. The latter exemplifies common use-cases in immunopeptidomics, particularly when identifying novel targets of interest.

As database size increases, most software packages reported increase number of candidate PSMs (at 100% PSM FDR filtering), except for PEAKS, which surprisingly exhibits a decrease (Fig. 5). This unexpected trend persists despite larger databases containing a superset of sequences from smaller ones, which should theoretically allow for more comprehensive peptide-spectrum matches. The cause remains undetermined despite consultation with Bioinformatics Solutions Inc. With 1% PSM FDR filtering, the results obtained with the largest database (UniProt + transcriptome + *de novo*) saw the largest reduction, to the point where the number of target identifications is the lowest for all software. Furthermore, we observed that the FDR cutoff value generally becomes stricter with larger databases (Supplemental Table S5). However, this is not a perfect correlation, indicating that other aspects play a role here.

When expanding the databases, especially when including sequences derived from DNTA into the database, we observed a high proportion of candidates PSMs derived from those in FragPipe, MHCquant and MaxQuant (Fig. 5*A* lighter shaded area). However, most of these PSMs are excluded upon FDR filtering (Fig. 5*C*).

The UniProt reference database consistently yielded the highest number of FDR filtered peptidoforms identifications for the PEAKS software, while the inclusion of the transcriptome resulted in most identification for the other three software. The addition of sequences from the DNTA data resulted in the lowest number of identifications. This indicates that addition of sequences of interest requires careful consideration to avoid overly expanding the search space, which may negatively affect the results.

DNA/RNA sequencing enables detection of peptide variants absent from reference databases. Single nucleotide variants, the most prevalent class of genetic variants, involves a single nucleotide change that may correspond to a single amino acid substitution resulting in a single amino acid variant (SAV). We evaluated the ability of the software to distinguish SAVs through the identification of pairs of SAV and its corresponding reference. All tools successfully detected such pairs, with both PEAKS software reporting both the highest number as well as fractions of this type of variant, especially when the DNTA were included (Fig. 5*D* both lighter shaded areas). However, many identified were leucine/isoleucine substitutions (Fig. 5*D* lightest shaded area) —indistinguishable by mass—suggesting that a single spectrum may match multiple peptide candidates, depending on how results are reported. This observation raises questions about whether such cases constitute distinct identifications. In PEAKS 11 Online, eight out of 13 and 32 out of 44 peptides are “IL variants” (for + transcriptome and + *DNTA* respectively). PEAKS X Pro follows a similar trend with 5/10 and 20/29 peptides. MHCquant has 4/10 and 8/20, MaxQuant has 2/3 and 2/2 and FragPipe has 0/5 and 3/16 IL variants. From this, PEAKS software should be the method of choice when “I/L variant” detection is relevant.

**Fig. 6:**
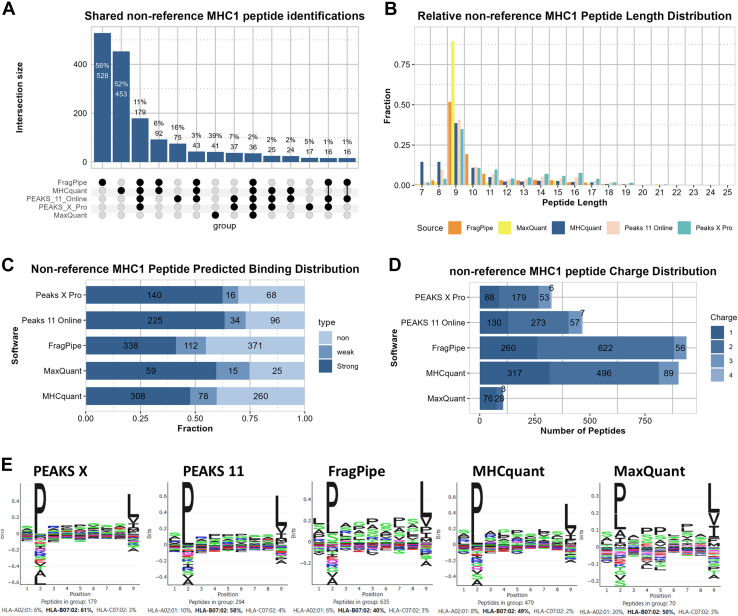
**Figures showing general properties of the non-reference peptide identifications**. *A*, upset plot showing the shared identifications. Groups smaller than 10 are omitted. *B*, length distribution plot of nonreference peptides. *C*, binding prediction of non-reference peptides. *D*, charge distribution of non-reference peptides. *E*, peptide motif of non-reference peptide identifications as reported by MhcVizPipe.

We were further interested in the non-reference identifications and examined their general properties (Fig. 6). Compared to the findings displayed in Figures 1 and 2, the results here show much lower consistency in shared peptides, length and predicted binding distribution and binding prediction. A notable difference in the binding motif is that the characteristic acidic amino acid signal at position 4, a prominent feature of HLA-A:02:01, was largely absent.

To further investigate how PSMs are affected by database size, we compared MS1 spectrum IDs between the UniProt-only database and the expanded UniProt + transcriptome + DNTA database (Supplemental Fig. S10). For both PEAKS software, many PSMs shifted to a “worse” category: PSMs that previously passed the FDR filter no longer did, and PSMs that did not pass the filter were no longer matched at all. Few new spectra were matched to peptides when using the UniProt + Transcriptome + DNTA database. In contrast, FragPipe and MHCquant matched many new spectra, explaining the gain in peptide identifications. Interestingly, MHCquant exhibited the largest changes among unique PSMs (those identified only by one of the search results) with most of these unique PSMs passing the FDR threshold. MaxQuant, unlike the other software, did not lose any spectrum IDs when switching to the larger database. The majority of spectra retained the same peptide assignment with high average scores. The spectrum that were either assigned to new sequences or left unassigned in one of the two search results (unique PSMs) generally had worse confidence scores, suggesting that high-confidence PSMs remain largely stable despite database expansion.

## Discussion

We compared five software packages based on four algorithms for immunopeptide identification from the optimized Thunder DDA-PASEF LC-MS data acquired in a timsTOF Pro2 mass spectrometer. We demonstrate that all evaluated software packages can identify the most evident subset of immunopeptides. PEAKS 11 with the build-in rescoring algorithm exhibited superior performance in immunopeptidome identification under standard operating conditions. FragPipe emerged as a viable non-commercial alternative, yielding comparable identification results.

Software selection depends on specific research needs. Although PEAKS 11 Online identified the highest number of peptides, its performance declined with increasing database size, potentially limiting its efficacy for analyses involving extensive reference databases. Correspondence with the PEAKS software staff revealed that this performance degradation may stem from the presence of similar sequences in larger databases. They caution against using reference databases containing numerous similar sequences, such as those incorporating novel SNPs and isoforms, as this may compromise their software's effectiveness^1^. In such cases, FragPipe is a good choice. On the other hand, PEAKS return the most I/L variant peptides, which may be important for studies emphasizing such targets.

There is ongoing discussion that applying FDR control on a per-group basis (grouped FDR control) can increase identifications within specific groups (39). In our view, this approach is most appropriate when there is a clear rationale for holding different subsets of identifications to separate statistical standards. For example, in multi-round searches where later rounds inherently operate on different quality spectra. This was not the case in our study, as all databases (both the entrapment approach and the database size comparison) were concatenated and searched in a single round.

The PEAKS 11th generation software offers an immunopeptide-specific workflow that employs a machine learning model tailored to immunopeptides. This particular approach was not incorporated in our analysis. Although this is a limitation of our study, here we focused on evaluating database search engines. Future research may benefit from a thorough examination of this workflow's performance and potential applications in immunopeptide analysis.

Machine learning-based rescoring functions have been shown to improve peptide identification under FDR filtering (40) in MaxQuant (19) and Peaks X Pro (14), which is also seen from our results comparing Peaks X Pro and Peaks 11 Online. The entrapment strategy demonstrated unbiased target-decoy performance across all software, including those utilizing deep learning algorithms. This finding is significant, addressing concerns about potential overfitting in deep learning-based methods. However, caution is warranted when using deep learning approaches due to inherent randomness, which may affect reproducibility. Thorough evaluation of deep learning models remains essential to identify potential biases that may impact result integrity.

Our findings contribute to the optimization of immunopeptidome analysis workflows and aid in software selection for future studies in this field. Especially low quantity targets can prove challenging for identification, making the correct choice of bioinformatics analysis essential. In the future we look towards data-independent acquisition (DIA) strategies, as search engines mature and AI driven technology are incorporated. DIA allows for the simultaneous acquisition of all precursor ions within a specified mass range, improving the depth and comprehensiveness of proteomic analysis, but at the same time generates more complex spectra that require much more sophisticated processing and bioinformatic analysis.

### Database Search Software Versions

Peaks X Pro: 10.6 build 20,201,221

Peaks Online 11: September 2023 version

Peaks Studio 11: build 20,230,202

FragPipe: 18.0/3.5/4.4.0/1.8.0 (FragPipe/MSFragger/Philosopher/IonQuant) – FragPipe 21.2-build03 was used for database size comparison.

MaxQuant: 2.4.4.0

MHCquant: 2.6.0dev

## Data Availability

The mass spectrometry immunopeptidomics and proteomics data have been deposited to the ProteomeXchange Consortium (41) via the jPOSTrepo partner repository (42) with the dataset identifiers PXD065501 for ProteomeXchange and JPST003768 for jPOSTrepo.

## Transcriptomic Data

The RNA-seq data for this study have been deposited in the European Nucleotide Archive (ENA) at EMBL-EBI under accession number PRJEB98909 (https://www.ebi.ac.uk/ena/browser/view/PRJEB98909).

## Supplemental Data

This article contains supplemental data.

## Conflictof Interests

The authors declare no competing interests.
