# Supplementary material for: Benchmarking Software for DDA-PASEF Immunopeptidomics
Source: Mol Cell Proteomics. 2025 Dec 19;25(4):101492. doi: 10.1016/j.mcpro.2025.101492 (PMC13085059; doi:10.1016/j.mcpro.2025.101492)
Supplement: Supplementary Data 1 [file mmc1.pdf]

Benchmarking Software for DDA-PASEF Immunopeptidomics

Authors:

Yannic Chen, Annica Preikschat, Annette Arnold, Riccardo Pecori, David Gomez-Zepeda, Stefan Tenzer

Content:

Figure S1 2
Table S1 3
Table S2 4
Figure S2 5
Figure S3 7
Figure S4 8
Figure S5 9
Table S3 10
Figure S6 11
Figure S7 12
Figure S8 13
Figure S9 16
Table S4 18
Table S5 19
Figure S10 20

Settings	MHC1	MHC2	MHC1 Boost	MHC2 Boost
6-45 length, 2 max mods, no boost	0 19154 0<br>Studio Online	0 27306 0<br>Studio Online	138 21545 210<br>Studio Online	300 29956 261<br>Studio Online
20ppm precursor, 0.05da, no boost	0 17166 0<br>Studio Online	0 25504 0<br>Studio Online	172 18903 141<br>Studio Online	237 27716 233<br>Studio Online
6-45 length, no boost	0 19165 0<br>Studio Online	0 27556 0<br>Studio Online	221 21606 212<br>Studio Online	308 30145 359<br>Studio Online

Figure S1 *Venn Diagram to compare the number of identification and overlap between PEAKS 11 Online and PEAKS 11 Studio using various settings and with or without deep learning boost*

PTM	MHC1				
	PEAKS X Pro	PEAKS 11 Online	FragPipe	MHCquant	Maxquant
None	15781	17476	15499	14733	5958
Oxidation	1493	1702	1805	1581	461
Cysteinylation	513	564	532	42	203
Acetylation	304	360	374	312	103
Acetylation; Oxidation	105	110	52	38	33
Cysteinylation; Oxidation	21	26	27	26	8
Acetylation; Cysteinylation	14	13	12	0	7
Acetylation; Cysteinylation; Oxidation	1	1	1	0	1

PTM	MHC2				
	PEAKS X Pro	PEAKS 11 Online	FragPipe	MHCquant	Maxquant
None	22537	24309	22923	21256	10991
Oxidation	2757	3043	2999	2778	893
Cysteinylation	623	734	653	730	238
Acetylation	539	641	562	52	156
Acetylation; Oxidation	94	122	63	33	24
Cysteinylation; Oxidation	58	70	60	83	13
Acetylation; Cysteinylation	11	14	10	0	7
Acetylation; Cysteinylation; Oxidation	3	3	3	0	3

Table S1 *Table showing the distribution of PTMs for MHC1 (top) and MHC2 (bottom) peptidoforms. Peptides with multiple different PTMs are counted separately. PTMs correspond to methionine oxidation, Protein N-term acetylation, and cysteine cysteinylation.*

MHC1

Software	Number of 8-13mer peptidoform identification at 1% PSM FDR		Difference in identifications
	Lenient setting (15ppm, 0.03da, max mass = 5000da)	Strict setting (10ppm, 0.02da, max mass = 1700da)	
PEAKS X Pro	13863	13880	+17
PEAKS 11 Online	15417	15185	-232
FragPipe	14182	13928	-254
MHCquant	12988	14190	+1202
MaxQuant	5032	3299	-1733

MHC2

Software	Number of 15-25mer peptidoform identification at 1% PSM FDR		Difference in identifications
	Lenient setting (15ppm, 0.03da, max mass = 5000da)	Strict setting (10ppm, 0.02da, max mass = 2900da)	
PEAKS X Pro	14635	14515	-120
PEAKS 11 Online	15971	17171	+1200
FragPipe	15819	16020	+201
MHCquant	14208	14690	+482
MaxQuant	7627	6248	-1379

Table S2 *Table showing the number of identifications between strict and lenient setting for MHC1 and MHC2 peptides.*

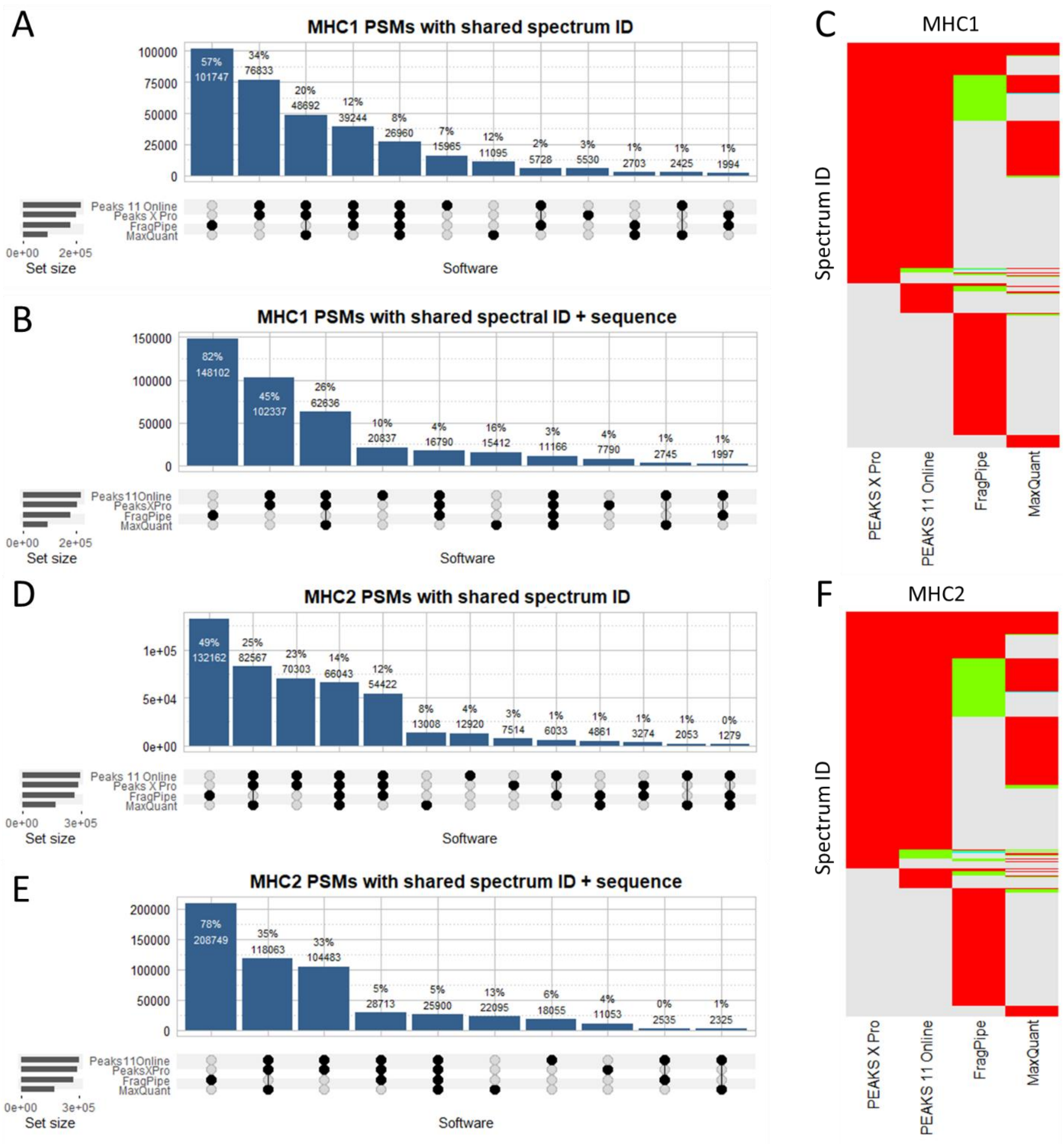

Figure S2 *Comparison of peptide identification across software at the PSM level. A) Upset plot of shared spectrum IDs of MHC1 peptides. B) Upset plot of shared spectrum IDs + sequence match of MHC1 peptides. C) Heatmap of spectrum IDs used by each software. Every row is a spectrum ID (duplicates are possible some software can report multiple matches for a single spectrum ID). Same colors in each row represent the same sequences that have been matched to the spectrum ID. D) Upset plot of shared spectrum IDs of MHC2 peptides. Grey color indicate that the spectrum ID was not matched to a sequence by the software. E) Upset*

plot of shared spectrum IDs + sequence match of MHC2 peptides. F) Heatmap of spectrum IDs for MHC2. All Upset-plot have a minimum group size of 1000.

A

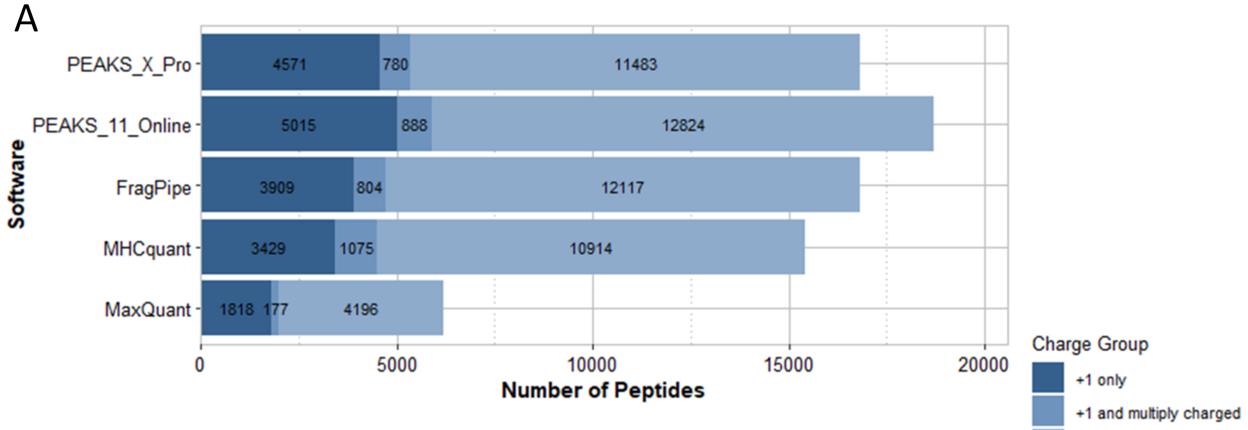

B

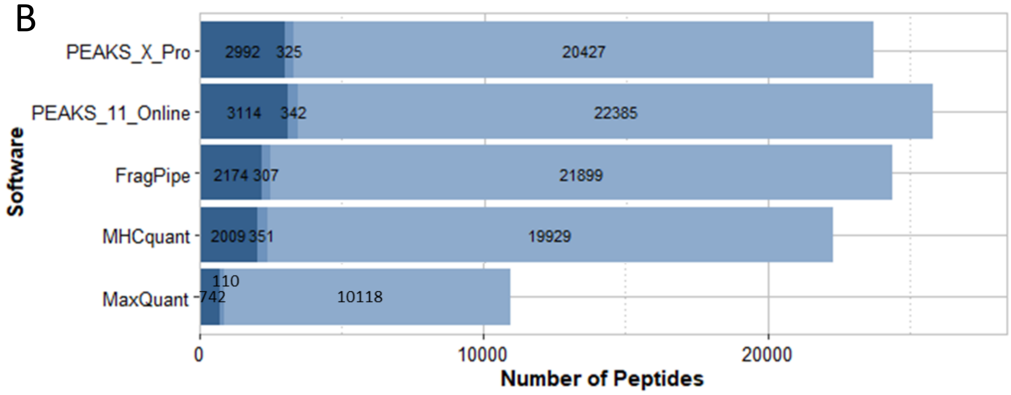

C

MHC1	Charge State Combinations									
	**1**	**1;2**	**1;2;3**	**1;3**	**2**	**2;3**	**2;3;4**	**3**	**3;4**	**4**
Peaks X Pro	4571	774	6	0	10056	424	7	945	25	25
Peaks 11 Online	5015	880	8	0	11216	478	7	1065	27	30
FragPipe	3909	799	5	0	10573	451	0	1093	0	0
MHCquant	3429	1061	12	2	9548	481	0	885	0	0
MaxQuant	1818	177	0	0	3785	90	2	310	5	4

D

MHC2	Charge State Combinations									
	**1**	**1;2**	**1;2;3**	**1;3**	**2**	**2;3**	**2;3;4**	**3**	**3;4**	**4**
Peaks X Pro	2992	323	2	0	11271	2854	88	5805	278	124
Peaks 11 Online	3114	339	3	0	12194	3074	114	6562	291	140
FragPipe	2174	305	2	0	11985	3056	0	6858	0	0
MHCquant	2009	348	3	0	10823	3018	0	6088	0	0
MaxQuant	742	110	0	0	6227	965	9	2804	78	35

Figure S3 *Bar plots showing the distribution of singly (+1 only) and multiply charged peptides (multiply charged only), as well as those peptides found as both singly and multiply charged (+1 and more) for MHC1 (A) and MHC2 (B) peptides. The detailed number of peptides found with all the charge state combinations are displayed in the tables for MHC1 (C) and MHC2 (D).*

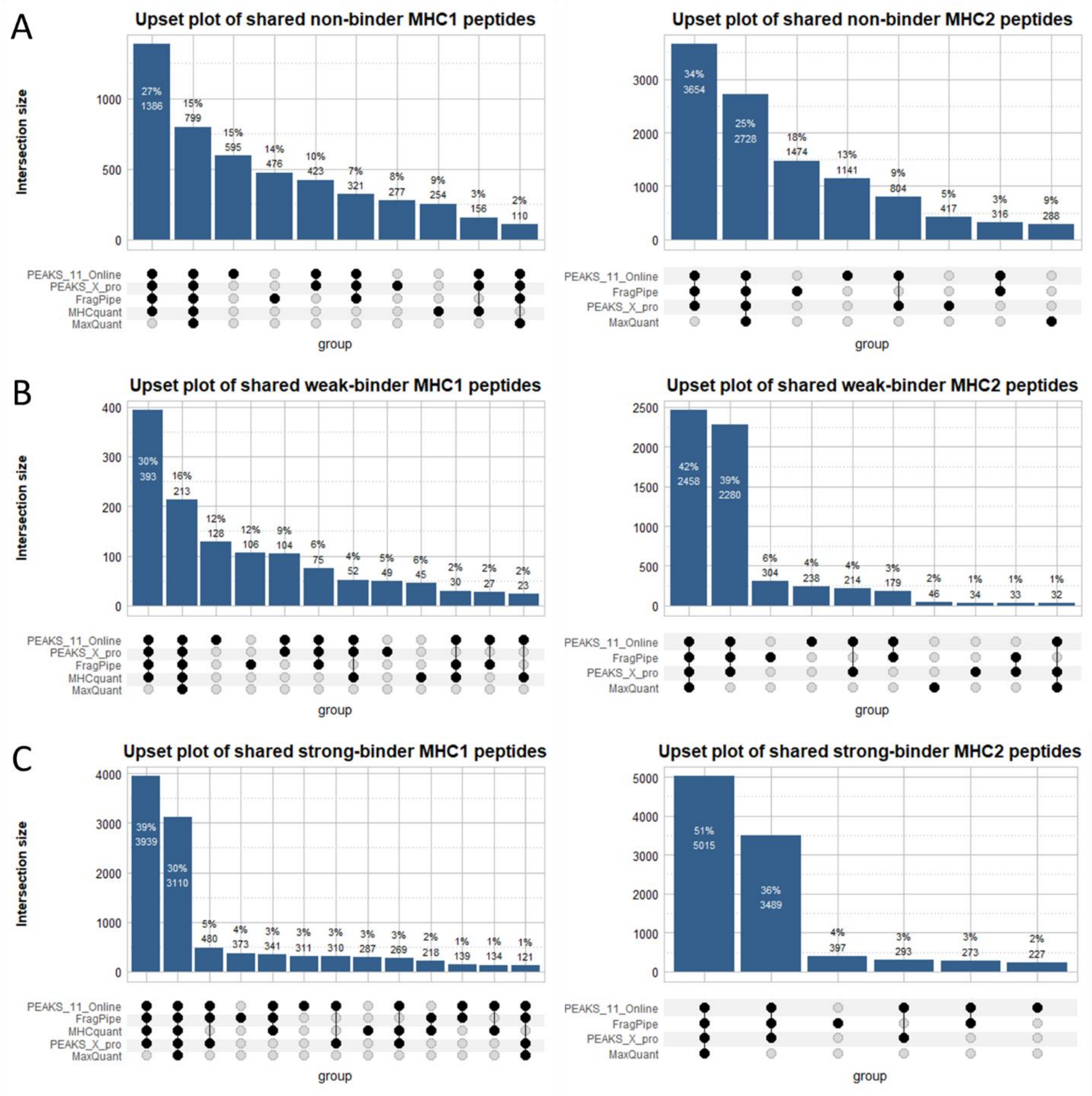

Figure S4 *Upset plot showing the number of shared predicted non-binding (A), weak-binding (B) and strong binding (C) MHC1 (left) and MHC2 (right) peptides. The minimum group size is set to 100 for all but the weak-binding peptides where the minimum group size is set at 20.*

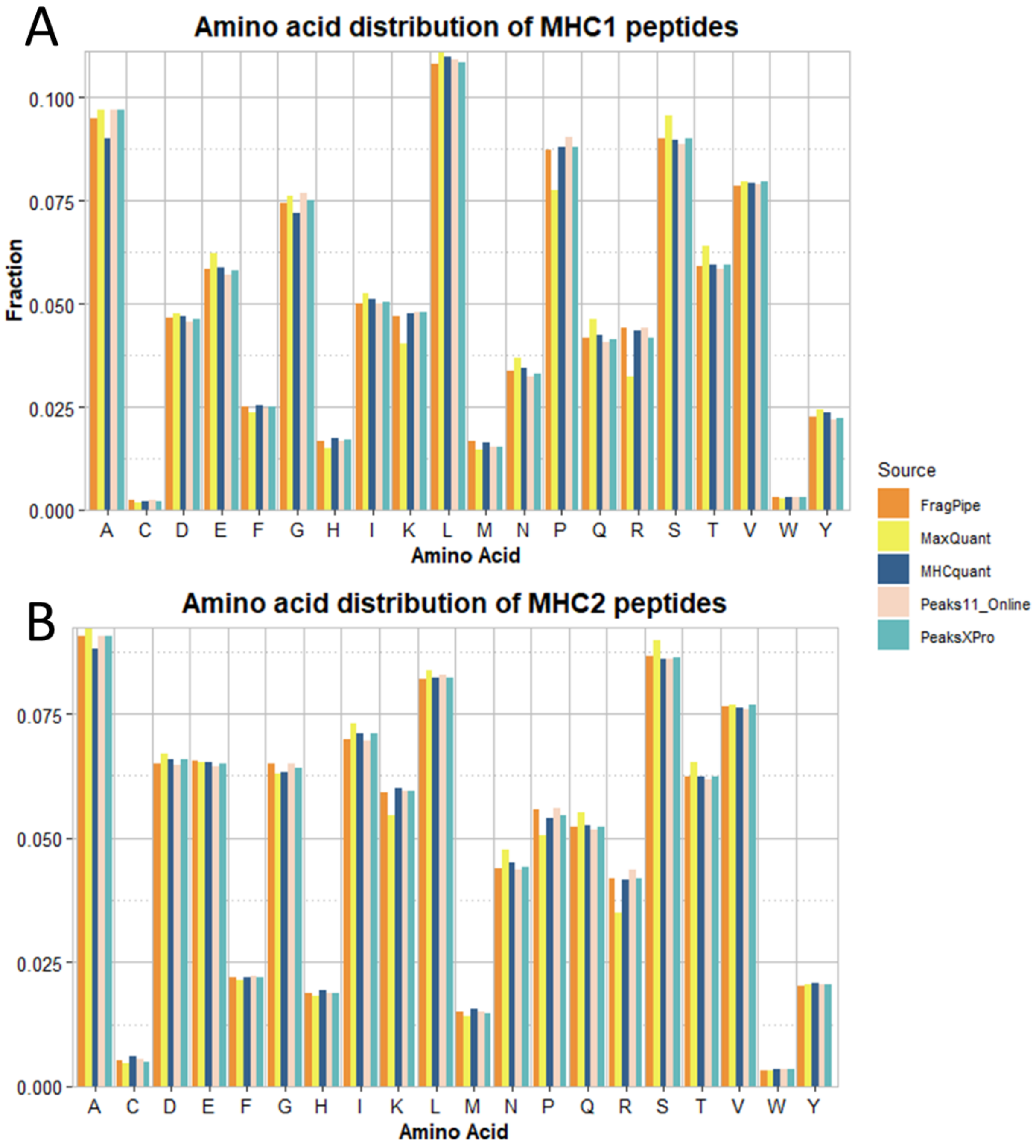

Figure S5 *Bar chart showing the distribution of amino acid of the identified peptides for each software for MHC class 1 (A) and class 2 (B).*

**MHC1**	**Peaks 11 Online**	**Peaks X pro**	**FragPipe**	**MaxQuant**	**MHCquant**
Aromatic content					
Charged residues				Depleted.	
Positively charged	Enriched.			Depleted.	
Negatively charged	Depleted.			Enriched.	
Polar (Zimmerman)				Depleted.	Enriched.
Hydrophobic (Eisenberg)	Enriched.				Depleted.
Hydrophobic (K-D)					
Hydrophobic (F-P)					
Exposed (Janin)					Enriched.
Flexible (Vihinen)				Depleted.	
High interface prop. (J-T)				Depleted.	Enriched.
High solvation poten. (J-T)					Depleted.
Frequent in alpha hel. (N)					
Frequent in beta struc. (N)	Depleted.			Enriched.	Enriched.
Frequent in coils (N)					
High linker propensity (G-H)				Depleted.	Enriched.
Disorder promoting (Dunker)	Enriched.			Depleted.	Depleted.
Order promoting (Dunker)				Enriched.	Enriched.
Bulky (Zimmerman)					
Large (Dawson)				Depleted.	Enriched.

**MHC2**	**Peaks 11 Online**	**Peaks X pro**	**FragPipe**	**MaxQuant**	**MHCquant**
Aromatic content					
Charged residues				Depleted.	
Positively charged	Enriched.			Depleted.	
Negatively charged					
Polar (Zimmerman)				Depleted.	Enriched.
Hydrophobic (Eisenberg)					
Hydrophobic (K-D)				Enriched.	
Hydrophobic (F-P)					
Exposed (Janin)					
Flexible (Vihinen)				Depleted.	
High interface prop. (J-T)					Enriched.
High solvation poten. (J-T)					
Frequent in alpha hel. (N)					
Frequent in beta struc. (N)				Enriched.	
Frequent in coils (N)					
High linker propensity (G-H)				Depleted.	
Disorder promoting (Dunker)	Enriched.		Enriched.	Depleted.	Depleted.
Order promoting (Dunker)				Enriched.	
Bulky (Zimmerman)				Enriched.	
Large (Dawson)				Depleted.	Enriched.

Table S3 *Results from Composition Profiler to determine property bias of identified peptides from each software. The sum of all identifications from all software were used as background. Grey indicate not significant. p < 0.005 is used as significance threshold.*

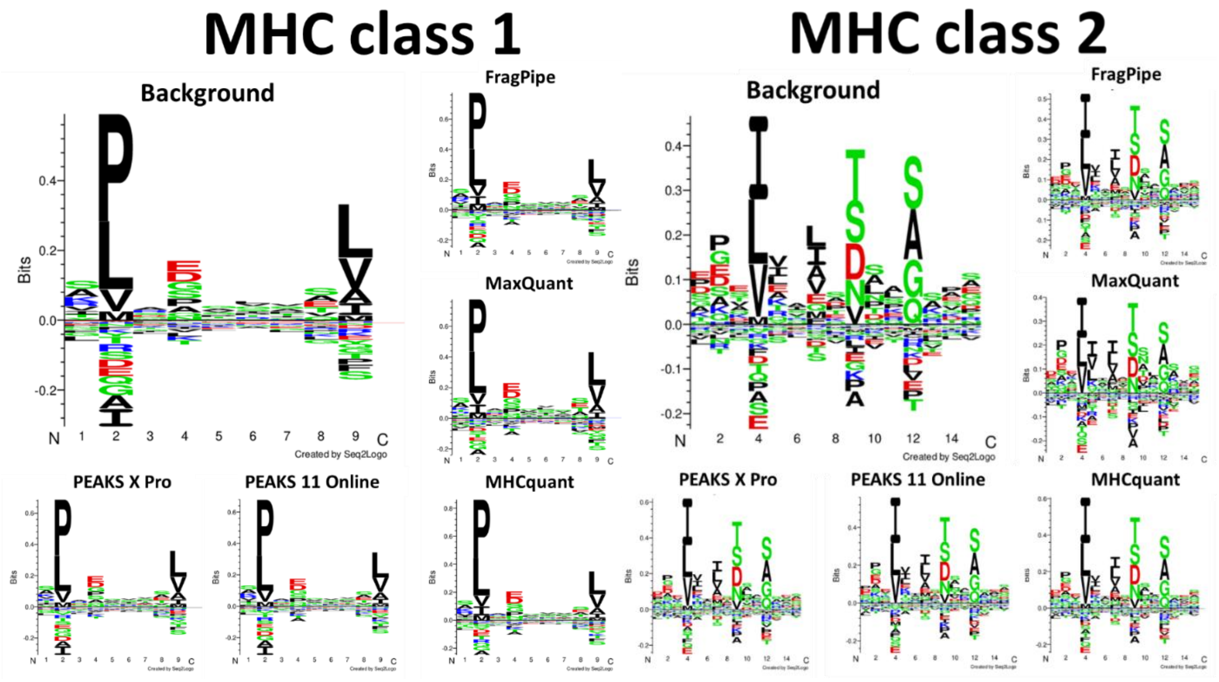

Figure S6 *Unsupervised Gibbs clustering of motifs for MHC class 1 (Top) and MHC class 2 (Bottom) peptide identifications from each software. Background is the combination of peptide identifications from all software. For MHC1, a motif length of 9 amino acids was used. For MHC class 2, 15 amino acid motif length was set.*

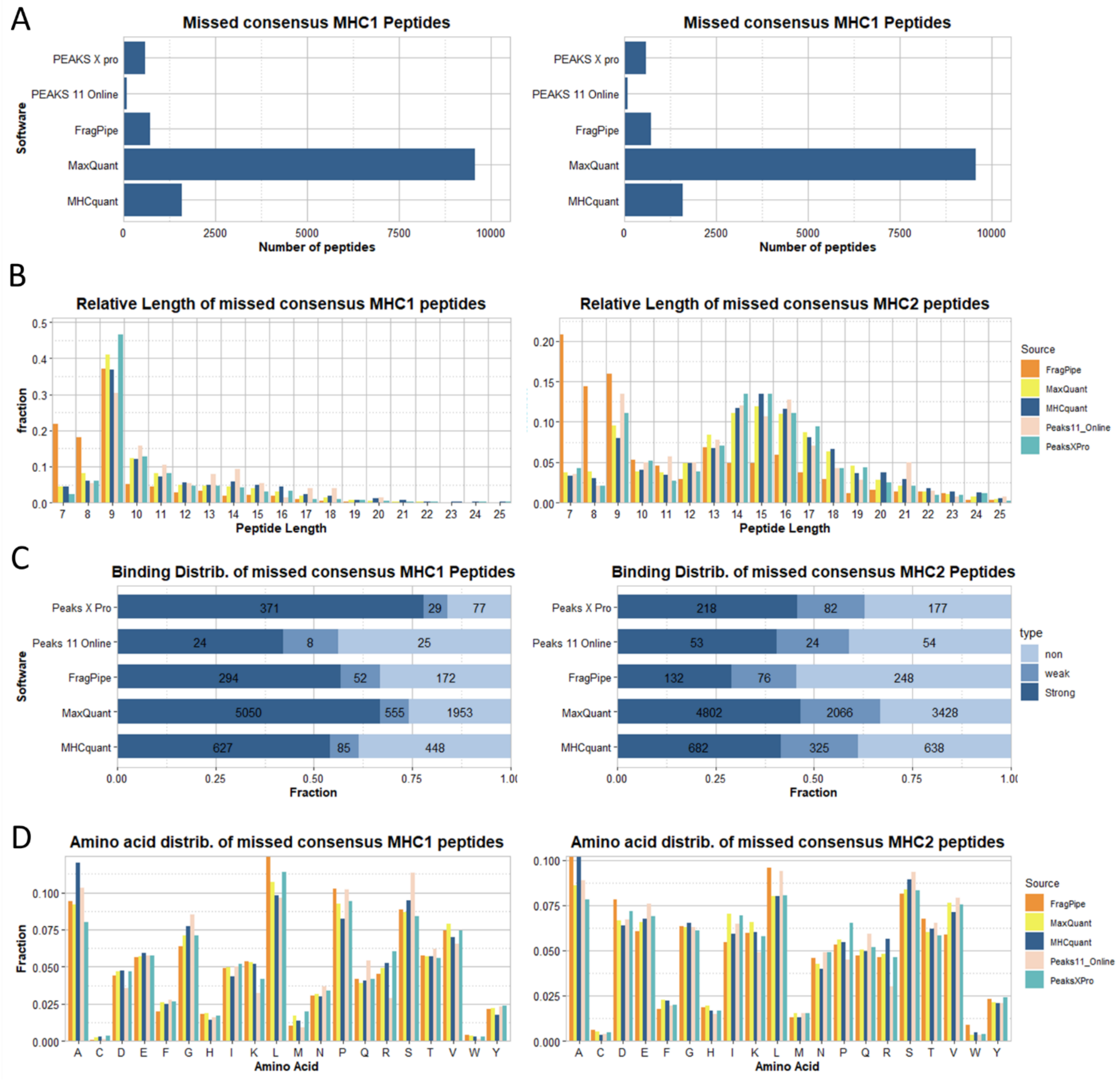

Figure S7 *Figures showing the properties of the consensus peptides (identified by at least three database search engines) missed by each software. A) Bar plot showing the number of consensus peptides not found by specific software. B) Length distribution plot of missed consensus peptides by each software. C) Binding strength distribution of missed consensus peptides for each software. D) Amino acid distribution of missed consensus peptides for each software.*

A

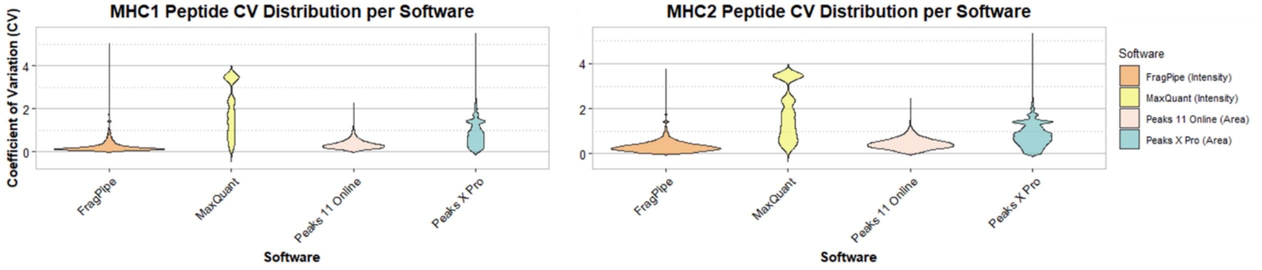

B

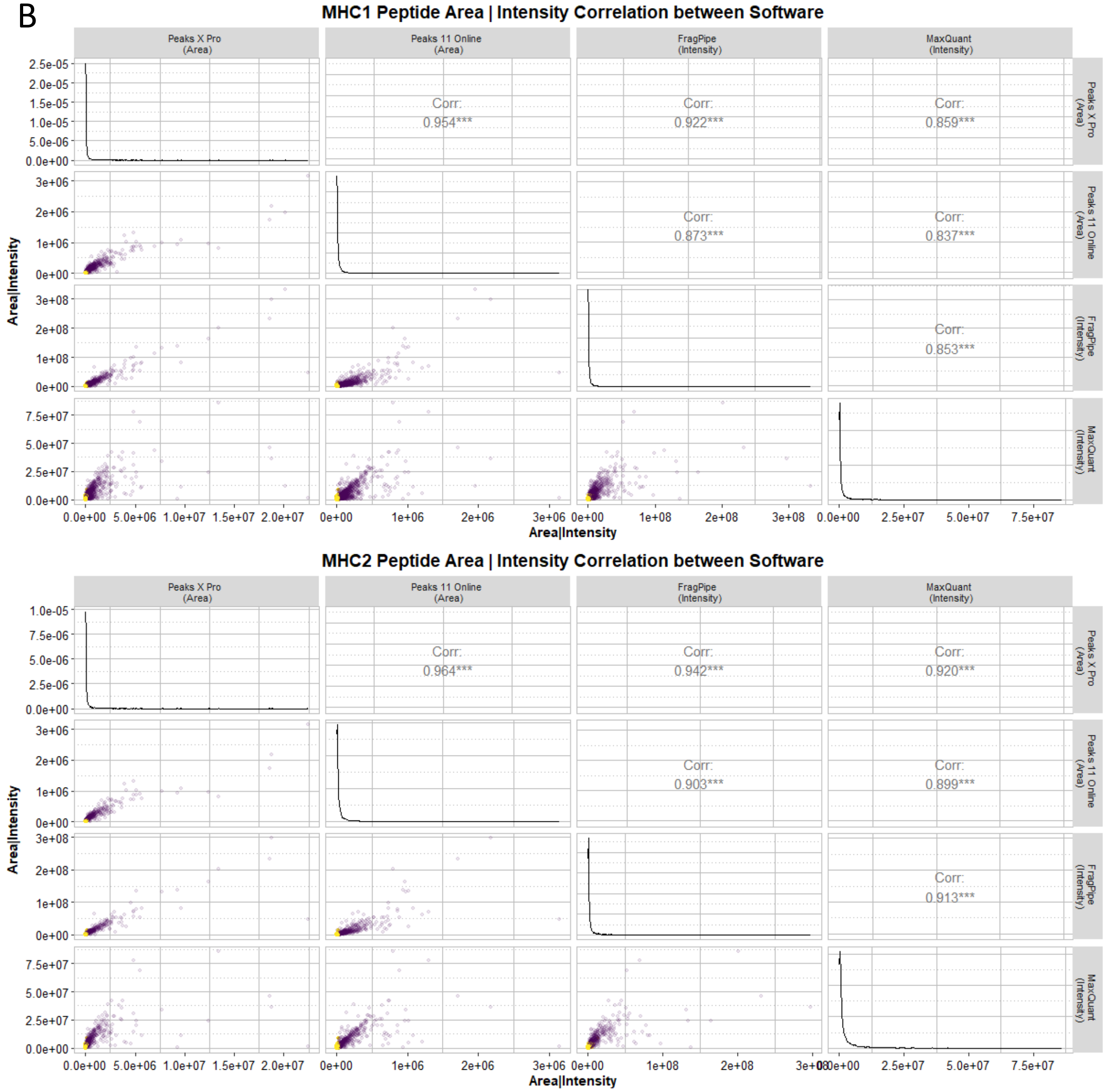

Figure S8 *Peptide quantification comparison between samples and between software. For the PEAKS software, area is used as quantification, while for FragPipe and MaxQuant, intensity was used. MHCquant quantification was disabled due to software*

problems. A) Coefficient of variation distribution between samples for each software. B) Paired scatterplot comparison between software. In case intensity/area are reported for each sample, these are summed to return a single value for a single peptide. Values at the top half indicate spearman rank coefficient.

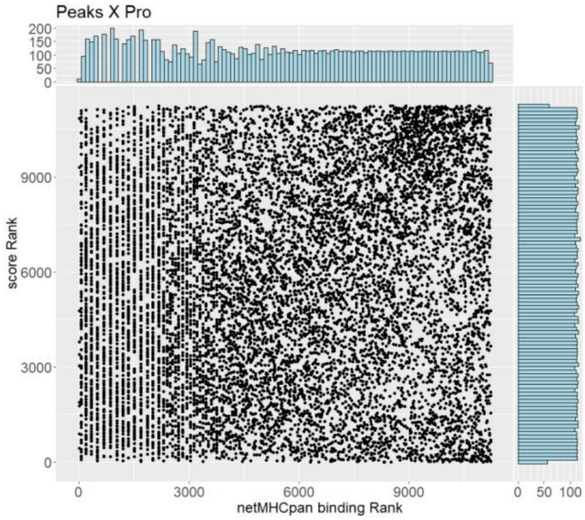
Peaks X Pro
score Rank
netMHCpan binding Rank
0
3000
6000
9000
0
50
100
150
200

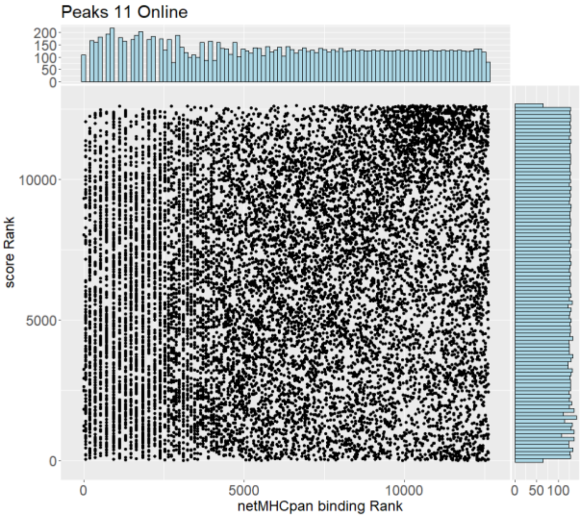
Peaks 11 Online
score Rank
netMHCpan binding Rank
0
5000
10000
50
100
150
200

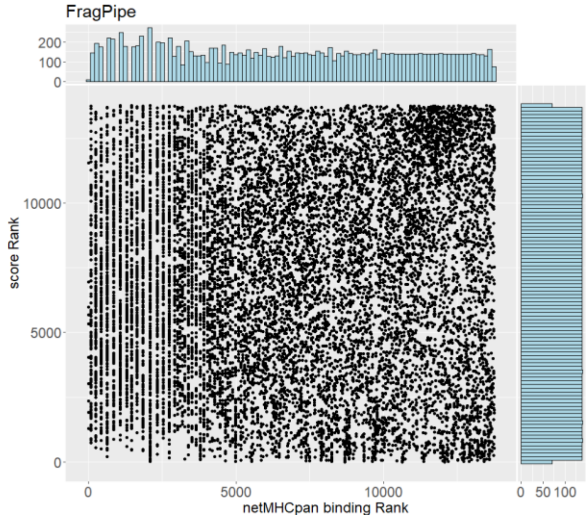
FragPipe
score Rank
netMHCpan binding Rank
0
5000
10000
100
200

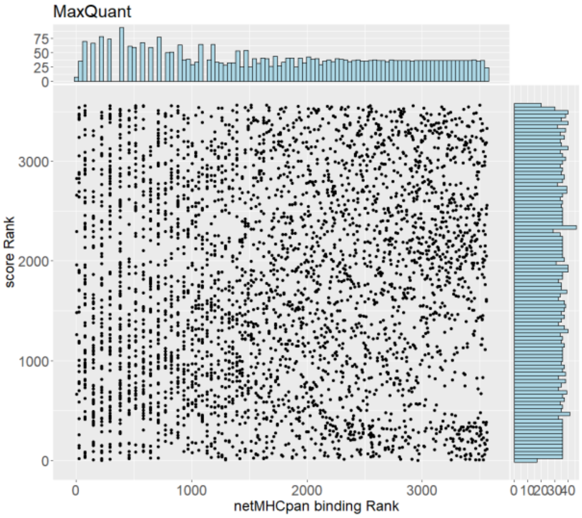
MaxQuant
score Rank
netMHCpan binding Rank
0
1000
2000
3000
25
50
75

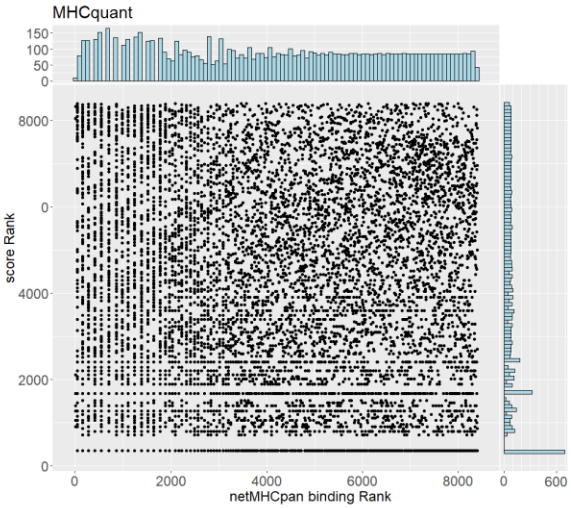
MHCquant
score Rank
netMHCpan binding Rank
0
2000
4000
6000
8000
600
50
100
150

	PEAKS X Pro	PEAKS 11 Online	FragPipe	MaxQuant	MHCquant
**Spearman correlation coefficient**	0.120	0.160	0.0598	0.089	0.0002

Figure S9 *Top) Scatterplot visualizing the peptide identification confidence as a function of their score rank for each software with their predicated binding strength from netMHCpan. Bottom) Table showing the spearman correlation for each of the scatterplots*

Number of unique peptides found at different PSM and peptide and peptidoform FDR (Decoy) on standard

MHC1	Peaks X pro	Peaks 11 Online	FragPipe	MHCquant	MaxQuant
1% PSM	16834 (2.7% peptidoform)	18727 (3.2% peptidoform FDR)	16830 (1.5% peptidoform)	15418	6191 (2.6% peptidoform)
1% peptide	13482	14719	13056	n/a	3812
5% peptide	16797	18110	16531	n/a	7356
10% peptide	19499	20091	18930	n/a	10129
1% peptidoform	14890	16269	14482	13765	4231
5% peptidoform	18506	19799	18226	17745	8389
10% peptidoform	21539	21986	20832	19582	11459

Number of unique **peptides** above 1% **peptide** FDR when using Decoy or ARATH as false positive (value in brackets denote score cutoff)

MHC1	Peaks X pro	Peaks 11 Online	FragPipe	MHCquant	MaxQuant
Decoy on std	13482 (21.85)	14719 (70.71)	13056 (0.9916)	n/a	3812 (0.01277)
Decoy on mixed	12987 (19.48)	14345 (68.1)	12912 (0.9840)	n/a	2756 (0.01272)
Arath on mixed	14555 (15.34)	15802 (49.16)	13632 (0.9750)	13647 (0.00506)	3792 (0.02090)

Number of unique peptides found at different **peptide** FDR (ARATH)

MHC1	Peaks X pro	Peaks 11 Online	FragPipe	MHCquant	MaxQuant
1% peptide	14555	15802	13632	13647	3792
5% peptide	21686	19333	17118	16539	8273
10% peptide	36216	23867	20066	18748	11732

Number of unique **peptidoform** above 1% **peptidoform** FDR when using Decoy or ARATH as false positive (value in brackets denote score cutoff)

MHC1	Peaks X pro	Peaks 11 Online	FragPipe	MHCquant	MaxQuant
Decoy on std	14890 (21.44)	16269 (68.30)	14482 (0.9907)	13765 (0.00995)	4231 (0.01407)
Arath on mixed	15914 (16.65)	17289 (47.68)	15077 (0.9730)	14944 (0.005373)	4302 (0.02351)

Number of unique peptides found at different **peptidoform** FDR (ARATH)

MHC1	Peaks X pro	Peaks 11 Online	FragPipe	MHCquant	MaxQuant
1% peptidoform	15914	17289	15077	14944	4302
5% peptidoform	23671	21024	18791	18151	9327
10% peptidoform	39894	26221	22067	20492	13189

Table S4 *Tables summarizing the number of identified MHC1p (and cutoff score) from each software at various FDR and with various FDR strategies: PSM, peptide and peptidoform. Peptides are defined as sequences with the PTMs removed, while peptidoforms retain the PTMs. The database used for these results are either the standard database (std) or the Arabidopsis entrapment database (mixed). False positives used for calculating the FDR is either decoy or Arabidopsis thaliana (Arath).*

	UniProt	UniProt + transcriptome	UniProt + transcriptome + DNTA
Peaks X Pro (higher = stricter)	15.97	18.06	18.10
Peaks 11 Online (higher = stricter)	24.38	24.25	31.21
FragPipe (higher = stricter)	0.9214	0.9226	0.9256
MaxQuant (lower = stricter)	0.035702	0.036796	0.026930
MHCquant (lower = stricter)	0.00999848	0.00999521	0.00999834

Table S5 *Tables showing the 1% PSM FDR cutoff value for all software. Higher = stricter: A higher value indicates greater confidence in the identification, therefore having a cutoff at a higher value is considered stricter. Likewise, for lower = stricter. DNTA, de novo transcriptome assembly.*

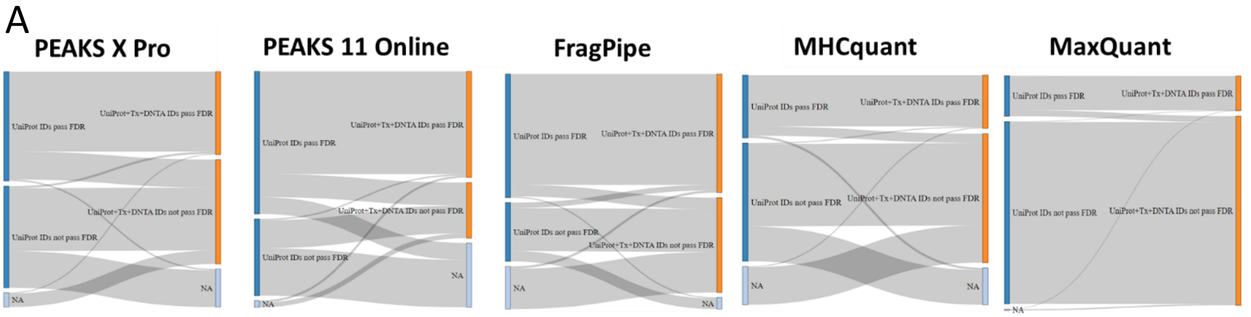

B

PSM		PEAKS X Pro	PEAKS 11 Online	FragPipe	MHCquant	MaxQuant
Same sequence	Number	243899	224150	241283	306197	521600
	UniProt mean score	26.91	72.45	0.90	0.15	0.447
	UniProt + Tx + DNTA mean score	22.90	56.67	0.90	0.14	0.447
Different sequence	Number	88731	18675	17829	114120	11701
	UniProt mean score	12.05	35.16	0.51	0.37	0.678
	UniProt + Tx + DNTA means core	10.32	23.08	0.45	0.34	0.678
Unique PSMs	Number	101065	111144	82258	219606	3211
	UniProt mean score	7.46	17.92	0.47	0.37	NA
	UniProt + Tx + DNTA mean score	8.64	25.02	0.266	0.35	0.643

Figure S10 *Changes in Spectra ID matching between results generated using the UniProt only database and the UniProt + transcriptome + DNTA. A) Sankey Plot visualizing the changes to the reported Spectrum ID used for PSMs between the two database search results. Left side of the Sankey plot refer to all spectrum IDs used by the search results with UniProt only and the right side of the Sankey plot refer to all spectrum IDs used by the search results with Uniprot + transcriptome + DNTA. NA corresponds to spectra not matched using the corresponding database. B) Table showing the number of Spectra ID that keep the same Peptide match (same sequence), that have been assigned a different sequence (different sequence) and that are only reported by one of the database search results (unique PSMs). The mean confidence score from both database search results is reported for each of these groups.*
